# Anticancer Activity of Two Novel Hydroxylated Biphenyl Compounds toward Malignant Melanoma Cells

**DOI:** 10.3390/ijms22115636

**Published:** 2021-05-26

**Authors:** Marina Pisano, Maria Antonietta Dettori, Davide Fabbri, Giovanna Delogu, Giuseppe Palmieri, Carla Rozzo

**Affiliations:** 1 Institute for Genetic and Biomedical Research (IRGB), National Research Council of Italy (CNR), Traversa la Crucca 3, 07100 Sassari, Italy; marina.pisano@cnr.it (M.P.); giuseppe.palmieri@cnr.it (G.P.); 2 Institute of Biomolecular Chemistry (ICB), National Research Council of Italy (CNR), Traversa la Crucca 3, 07100 Sassari, Italy; mariaantonietta.dettori@cnr.it (M.A.D.); davidegaetano.fabbri@cnr.it (D.F.); giovannamaria.delogu@cnr.it (G.D.)

**Keywords:** melanoma, curcumin analogs, hydroxylated biphenyl, apoptosis

## Abstract

Melanoma, the deadliest form of skin cancer, is still one of the most difficult cancers to treat despite recent advances in targeted and immune therapies. About 50% of advanced melanoma do not benefit of such therapies, and novel treatments are requested. Curcumin and its analogs have shown good anticancer properties and are being considered for use in combination with or sequence to recent therapies to improve patient outcomes. Our group previously published the synthesis and anticancer activity characterization of a novel curcumin-related compound against melanoma and neuroblastoma cells (D6). Here, two hydroxylated biphenyl compounds—namely, compounds **11** and **12**—were selected among a small collection of previously screened C2-symmetric hydroxylated biphenyls structurally related to D6 and curcumin, showing the best antitumor potentiality against melanoma cells (IC_50_ values of 1.7 ± 0.5 μM for **11** and 2.0 ± 0.7 μM for **12**) and no toxicity of normal fibroblasts up to 32 µM. Their antiproliferative activity was deeply characterized on five melanoma cell lines by performing dose-response and clonal growth inhibition assays, which revealed long-lasting and irreversible effects for both compounds. Apoptosis induction was ascertained by the annexin V and TUNEL assays, whereas Western blotting showed caspase activation and PARP cleavage. A cell cycle analysis, following cell treatments with either compound **11** or **12**, highlighted an arrest in the G2/M transition. Taking all this evidence together, **11** and **12** were shown to be good candidates as lead compounds to develop new anticancer drugs against malignant melanoma.

## 1. Introduction

Recently, melanoma has become a major health and social problem due to its impressive increase in incidence in the Caucasian population [1,2]. It is estimated that, annually, around 100,000 new cases of cutaneous melanoma occur in the world (15% more than the previous decade) [3]; in Italy, about 14,900 new cases of cutaneous melanoma were estimated in 2020 (4% of all cancers), with an increase of 15% compared to 2011, making melanoma, respectively, the second (10%) and third (8%) most frequent neoplasia by incidence in men and women under the age of 50 [4]. Despite melanoma presenting an intrinsic propensity toward invasiveness and metastasis formation, the vast majority of increased incident cases are surgically removed at the early disease stage before tumor dissemination [5]. Therapy options for advanced/metastatic melanoma instead remain poor, and patients develop a strong resistance to almost all conventional chemotherapeutic treatments [6]. The degree of aggressiveness is even higher when melanoma interest mucosal surfaces [7].

Melanoma is derived from the malignant transformation of melanocytes through the sequential accumulation of various genetic molecular alterations, driving normal melanocyte to become malignant melanoma cells with the subsequent acquisition of metastatic capacity [8]. Genome-wide association studies over the last decade have been extremely useful in identifying several genetic alterations associated with cancer onset and progression [9]. Among them, those shown to play a main role in the pathogenesis of melanoma are (a) the oncogenic activation of the genes that control cell cycle progression, with a particular reference to the genes of the MAPK molecular pathways (that mainly includes *BRAF* and *NRAS*), (b) inactivation of the apoptosis and cellular senescence control mechanisms (apoptotic pathways), and (c) increase in the metastatic potential, through the alteration of the mechanisms controlling the anchorage-dependent growth, and the consequent induction of remote cell migration and dissemination [10]. All these pieces of evidence indicate the existence of a complex set of molecular mechanisms responsible for the control of melanocytic cell proliferation by balancing the induction (oncogenic) and suppression (tumor suppressive) activity of neoplastic transformation [11].

Melanoma is a heterogeneous neoplasm that is notoriously and markedly refractory to chemotherapy. Recently, a number of molecules with antitumor activity, targeting intracellular kinases involved in neoplastic pathogenesis, have begun to be used in clinical practice (targeted therapies). In particular, selective BRAF (vemurafenib, dabrafenib, and encorafenib) mostly administered in combination with MEK (cobimetinib, trametinib, and binimetinib) inhibitors, as well as the PI3K/AKT kinase pathway inhibitors, have shown promising results in clinical trials [12]. The use of immunotherapeutic agents (i.e., the immune checkpoint inhibitors), given in sequence or combination with targeted therapies, is now achieving satisfactory clinical outcomes in large fractions of patients presenting alterations in candidate target genes. Actually, about 50% of metastatic melanoma patients gain long-term benefits from immune and target therapies, while chemotherapy is indicated only for patients who do not respond to them [13,14]. Overall, one could state that *BRAF*-mutated melanoma patients may indeed benefit of an additional treatment opportunity represented by the targeted therapies. In other words, the therapeutic weaponry is more limited for patients with cutaneous melanoma lacking a *BRAF* mutation (about 50% of all cases) and for those with rare melanoma subtypes (e.g., uveal, acral, and mucosal). Moreover, about one-tenth of melanoma patients is innately resistant to therapy (primary resistance), whereas all the remaining ones become resistant following an initial response due to the activation of alternative proliferative pathways in the cancer cell (acquired resistance) [14,15]. Therefore, there remains a need to improve the outcomes for patients left the “orphans” of any therapy or for those who experience drug resistance, as well as to combine additional therapeutic compounds aimed at increasing the responsiveness to current treatments and, thus, at ameliorating the global prognosis [16].

For these reasons, many research groups keep investigating the search for new compounds with a wider spectrum of antiproliferative activities to be used in combination with or sequence to targeted therapies and immunotherapies [17]. Several compounds of natural origin have recently proven to exert promising activity [18,19,20,21]. These include curcumin and some of its analogs, which, besides the recognized antioxidant and anti-inflammatory properties, have demonstrated antitumor and proapoptotic effects on many cancer types [22,23,24,25,26,27,28]. Moreover, curcumin has already been used as an oral supplement in various dermatological conditions [29].

In the past years, our group has developed a new compound, a hydroxylated biphenyl, structural analog of curcumin, the α,β-unsaturated ketone called D6, for which an antitumor effect has been demonstrated at concentrations around 2 μM against melanoma and neuroblastoma, both in vitro and in vivo on animal models [30]. This compound was shown to act by inhibiting cell proliferation through cell cycle arrest in the G2-M transition and by inducing the apoptotic process through the involvement of the intrinsic pathway and activation of caspases. Noteworthy, the D6 compound showed low toxicity in the preclinical models. Expression profiling unveiled an interesting involvement of proapoptotic pathways for D6 [31,32] and, taking all our results together, we considered it an excellent candidate for developing a new multitarget therapeutic agent against melanoma. However, its activity needed to be improved. The low solubility of this compound in physiological solutions initially created difficulties in administering it to mice during the in vivo testing phase. Trying to overcome this problem, our group synthesized a battery of D6-like compounds to be tested for their biological activities, solubility, and bioavailability to optimize their anticancer potentiality [33].

**Figure 1:**
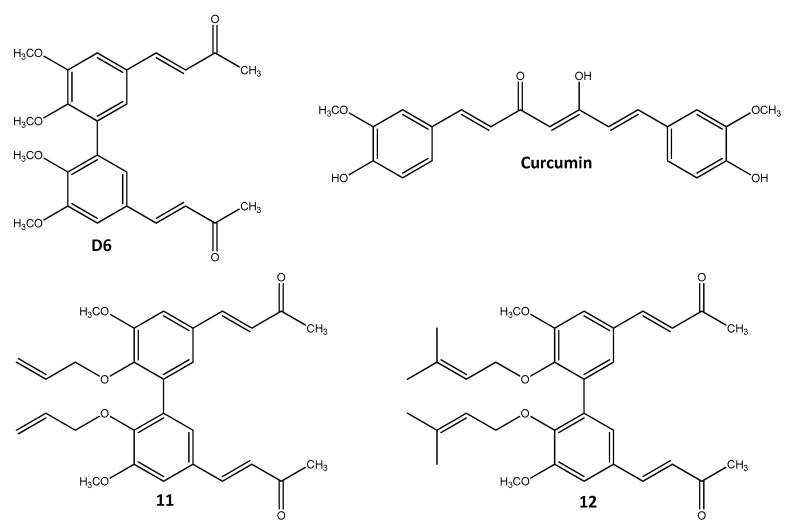
Chemical structures of **11**, **12**, D6, and curcumin.

Among the thirteen newly synthesized compounds, we selected two of them, **11** and **12** (Figure 1), showing the best antiproliferative activity on melanoma cells (IC_50_: 1.7 ± 0.5 μM and 2.0 ± 0.7 μM, respectively) and good stability and bioavailability compared to curcumin [33]. In this paper, we investigated their antitumor activity against melanoma cells in terms of both dose and time dependence and their capability of inducing apoptosis and interfering with cell cycle progression.

## 2. Results

Based on the preliminary data coming from an extensive screening of the biphenyl compounds [33] structurally related to compound D6 [30,31,32], we selected two hydroxylated biphenyl structures, compounds **11** and **12** (Figure 1), that showed interesting potential antitumor activity in melanoma cells (IC_50_ 1.7 ± 0.5 μM and 2.0 ± 0.7 μM, respectively). To verify this antitumor potentiality, we performed several biological assays, including proliferation assays, clonal growth assays, apoptosis assays, and a cell cycle analysis, on five melanoma cell lines representing different molecular patterns of melanoma, as described in the Materials and Methods section: CN-mel (CN), GR-mel (GR), A375, PR-mel (PR), and SK-mel (SK) [34].

### 2.1. Antiproliferative Activity

The efficacy of **11** and **12** against melanoma cell proliferation was preliminary confirmed by comparing their effects on two MM cell lines (CN and GR) and normal fibroblast BJ to that of D6 and curcumin. The results of such dose-response proliferation assays, reported in Figure S1 of the Supplementary Materials, clearly showed a stronger antiproliferative activity for all the three derivatives compared to curcumin (*p* < 0.001), confirming our previously published data [30,33].

**Figure 2:**
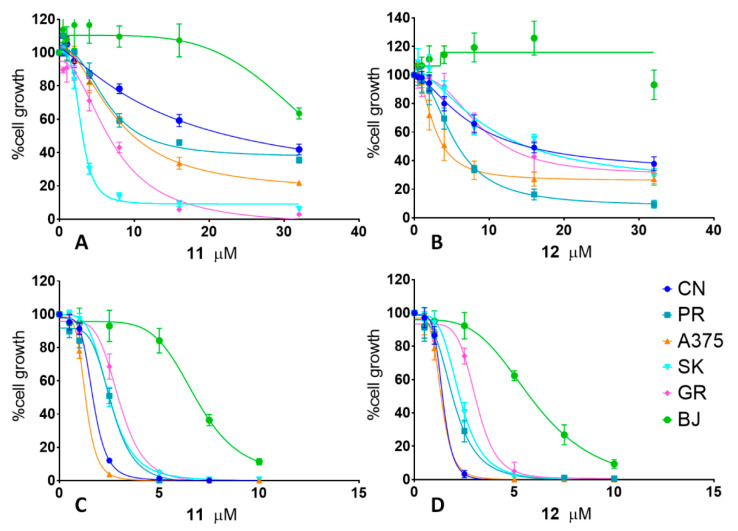
Antiproliferative activity: Cells were cultured with increasing concentrations (0.5–32 μM) of either **11** (**A**,**C**) or **12** (**B**,**D**) up to 24 h (**A**,**B**) or 72 h (**C**,**D**). Cell proliferation values were calculated as the growth percentages of treated cells compared to the untreated ones. Graphs represent the curves extrapolated [35] by the nonlinear regression of a variable slope (four parameters), with GraphPad Prism (v. 7.00) for the results of three experiments, each done in triplicate, ± the standard deviation, as described in Materials and Methods.

To better characterize the antiproliferative activity of the selected compounds, **11** and **12**, we first performed more dose-response proliferation assays on the five mentioned MM cell lines in parallel, with the normal fibroblast cell line BJ as the control. The cells were treated with increasing concentrations of either **11** or **12** up to 24 h (T24) or 72 h (T72). The results, reported in Figure 2, showed an effective, dose-dependent antiproliferative activity of the two compounds, **11** and **12**, on the melanoma cell lines while showing poor efficacy in the control cells (BJ fibroblasts). Indeed, in the T24 assays, all MM cell lines show a proliferation decrease trend, while doses up to 32 μM of **11**/**12** did not significantly inhibited fibroblast growth (Figure 2A,B). In longer treatments instead (T72; Figure 2C,D), some antiproliferative activity was observed on BJ fibroblasts starting from 5-µM treatments, although significantly lower than that of the MM cells (*p* < 0.001). These results confirmed the preliminary IC_50_ data reported in our previous study [33]. As a positive control for antiproliferative activity, all the cells were treated, in parallel, with 10-µM cisplatin, a traditional antitumor chemotherapeutic drug (Figure S2).

Based on these dose-response assays results, the IC_50_ values were calculated for all the cell lines treated with both compounds **11** and **12**, which are reported in Table 1. We observed a certain heterogeneity of the IC_50_ values for the different cell lines analyzed—in particular, for the T24 assays—while the T72 IC_50_ values became similar and significantly lower than those registered for the BJ fibroblasts (*p* < 0.001).

**Figure 3:**
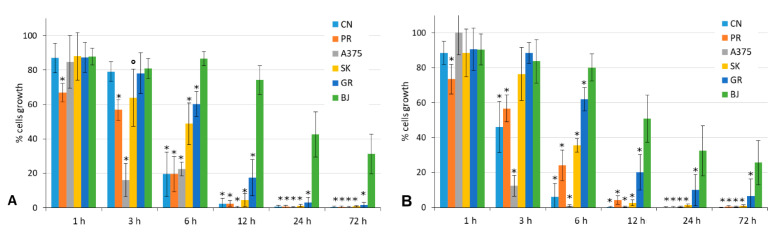
Time dependence. Drug wash-out assays assessed the impact of **11** and **12** time exposures on the cell proliferation of both MM cells and BJ fibroblasts. Cells were incubated with 5 μM of either **11** (**A**) or **12** (**B**) for the indicated times and then washed and cultured in drug-free medium up to 72 h. Cell proliferation was assessed by MTT assays as described. The results were derived from three different experiments, each done in triplicate, and expressed as the mean percentage of cell growth ± standard deviation. ° *p* ≤ 0.05, SK cell line vs. BJ; * *p* ≤ 0.001, MM cell line vs. BJ.

To test the time dependence and point out the efficacy and the long-term effects of the antiproliferative activity of **11** and **12** on the melanoma cells, we performed wash-out proliferation assays. Cells were grown in the presence of **11** or **12** for variable times, then washed and grown in complete culture medium up to 24 h (T24; Figure S3) or 72 h (T72; Figure 3). These assays allow us to determine the shortest treatment time necessary to obtain an effective antiproliferative activity on the tumor cells. They also evaluate the reversibility of the tested compound effect. Considering the IC_50_ values reported in Table 1, the cells were treated with a 5-μM dose of either the **11** or **12** compound for 6, 12, and 24 h (T24; Figure S3) and for 1, 3, 6, 12, 24, and 72 h (T72; Figure 3). The T24 point of the experiment (Figure S3) showed that the initial activity of **11** and **12** were already evident, especially on A375 cells, inhibiting the proliferation during short times of exposure (6 h) and still visible at the end of the experiment, a total of 24 h. The most significant results were obtained during the T72 point of the experiment. As shown in Figure 3A,B, some more sensitive cell lines showed the effect of both compounds after only 1–3 h of treatment (PR after 1 h and A375 and SK after 3 h), and even after the reconstitution of the complete medium, those cells did not reacquire the proliferation activity. After 6 h of treatment, all the MM cell lines were highly inhibited, while the BJ fibroblasts kept growing, and at the end of the 72-h experimental time, the MM cells showed 40–80% growth inhibition vs. about 10–20% inhibition shown by the BJ fibroblasts (*p* ≤ 0.001). After 12 h of treatment, at the end of 72 h, cell proliferation was almost abolished (0–20%) in all the MM cells, while, for the fibroblasts (BJ), it was only decreased to 74% by **11** and to 51% by **12** (*p* ≤ 0.001). These results demonstrate the high selectivity of compounds **11** and **12** toward tumor cells in treatments for up to 12 h of continuous exposure to the drug.

To better characterize the long-term effects of **11** and **12** on tumor growth arrest, we performed clonogenic survival assays in both liquid and semi-solid media. Each cell line was first characterized for its specific tumorigenic potential expressed as the percentage of cells able to grow clonally (Table 2A). Except for the GR cell line, which did not generate isolated colonies with both methods and was therefore considered potentially nontumorigenic, all the other MM cell lines showed tumorigenic capabilities. Each cell line demonstrated different behaviors of clonal growth: the A375 and PR cell lines did not show the capacity to develop single colonies in the liquid medium and, therefore, were grown only in the semi-solid medium, while SK did not grow in the semi-solid medium, and we only considered the results obtained in the liquid medium. CN was the only one that showed a clonal growth activity in both the liquid and solid media.

**Figure 4:**
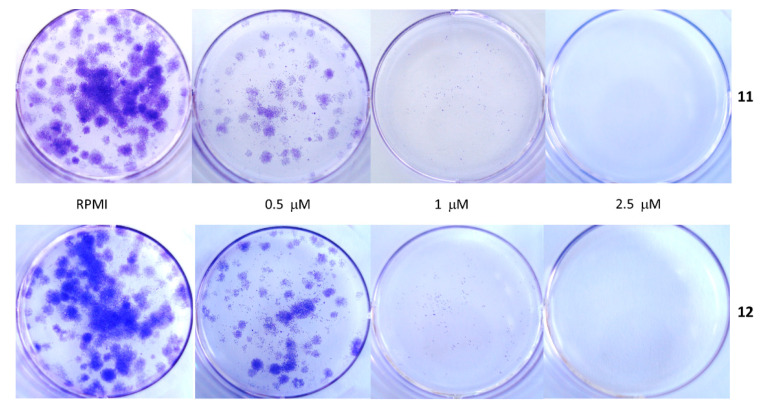
Clonal growth inhibition. Effects of **11** and **12** on the clonal growth of SK melanoma cells. Cells were suspended in a medium containing the indicated increasing concentrations of **11** and **12** for 4 h and then washed, seeded with a drug-free medium in a 12 well-plate, and grew up over 14 days. Cell colonies were stained with crystal violet, counted, and photographed as described [30].

To investigate the ability of our compounds to inhibit the tumor cell clonal growth, we treated these MM cell lines with increasing concentrations of either **11** or **12** (0.5–10 μM) periodically for 14 days, performing both continuous and wash-out treatments. The results of these experiments are summarized in Table 2B. Both compounds **11** and **12** were able to dose-dependently inhibit clonal tumor cell proliferation with all the MM cell lines tested. Their activity was comparable to that of the parent compound D6 [30]. Table 2 reports the minimal concentration of both compounds that inhibited the clonal growth of about 90–100% for each cell line, in consideration of the different methods used to perform the experiments. It can be noted that the PR and CN cell lines, which showed greater clonal growth capacity (9.86% and 9.61%, respectively), were more resistant to **11** and **12**, requiring doses of 10 and 5 μM, respectively, to completely inhibit the tumor cell growth in semi-solid media. Instead, A375 cells were the most sensitive; indeed, the 2.5-μM dose of **11** and **12** was sufficient to obtain the same result, according to its lower clonal growth ability (4.65%). In the liquid medium, the continuous exposure to each of the two compounds for 14 days was more effective in terms of the antitumorigenic activity (100% inhibition at concentrations of 2.5 μM and 1 μM for CN-mel with **11** and **12**, respectively). For SK, we obtained results only from the clonogenic assay inhibition performed in the liquid media, and Figure 4 shows an example of the dose-dependent inhibition of tumorigenicity by both **11** and **12** for this cell line. In the semi-solid medium, where the cells grew anchored to a 3D support, the concentrations that caused a total inhibition of the tumorigenic activity were raised to 5–10 μM. The wash-out experiments in semi-solid conditions again showed that the growth arrest caused by the treatments with **11** and **12** lasted over time. In fact, only 4 h of exposure to each compound inhibited the tumorigenicity that was maintained over a time of up to 14 days in the absence of the inhibitor and in a dose-dependent manner.

### 2.2. Apoptosis Induction

To determine whether the antiproliferative activity of **11** and **12** on melanoma cells was accompanied by the induction of apoptosis, we performed several assays that highlighted the different aspects of the apoptotic process: annexin V assays, caspases detection by Western blots, and TUNEL assays.

#### 2.2.1. Annexin V Assays

**Figure 5:**
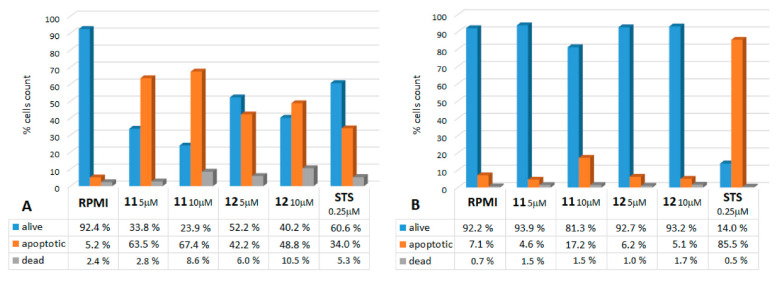
Apoptosis induction: annexin V assay. The graphs show the results on the (**A**) melanoma cells (SK) and (**B**) control fibroblasts (BJ) following a 16-h treatment with **11** and **12** at concentrations of 5 and 10 μM. The data represent the percentage of alive, apoptotic (early + late), and dead cells after 16 h from the administration of the compounds. Staurosporin (STS) at a 0.25-μM dose was used as an apoptosis inducer, the positive control.

In this assay, annexin V binds to phosphatidylserine externalized on the surface of apoptotic cells, which is considered an early event in the apoptotic process. The MM cell lines and BJ fibroblasts were treated with 5-μM and 10-μM doses of either **11** or **12** for 16 h, then processed as described in the Materials and Methods. These experiments highlighted the ability of compounds **11** and **12** to dose-dependently induce apoptosis in the MM cells. In Figure 5, the annexin V data from the SK cells (as an example among all five MM cell lines) treated with either **11** or **12** (Figure 5A) are compared to those obtained from the BJ fibroblasts, where this induction is barely visible (Figure 5B). In this particular case, the SK cell line was more sensitive to **11** than to **12**: at a concentration of 10 μM, **11** caused apoptosis in about 68% of the cells, compared to 49% caused by **12** at the same concentration. Noteworthy, the test showed that the effects of **11** and **12** were also stronger than that of 0.25-μM staurosporine (34% apoptotic cells), the proapoptotic agent used as a positive control of the experiments.

Table 3 summarizes the proapoptotic effects of **11** and **12** on all five MM cell lines and on the fibroblasts, reporting the percentage of Annexin V-positive cells resulting after the treatments. The MM cell lines showed heterogeneous responses, but still, apoptosis values were significantly higher with respect to those registered on normal BJ fibroblasts for all of them (*p* ≤ 0.01), with the exception of A375 with compound **11**. Unexpectedly, this MM cell line was shown to be less sensitive to the proapoptotic effect (19.2% **11** and 18.7% **12**), despite being the most affected by the antiproliferative and antitumorigenic activities, with the lowest IC_50_ values (2.9 μM and 3.8 μM for **11** and **12,** respectively, T24; see Table 1) among the five MM cell lines tested, while SK (67.1% **11** and 49.3% **12**) and PR (68.6% **11** and 33.7% **12**) were shown to be the most sensitive.

#### 2.2.2. Caspases and PARP Activation

Activation of aspartate-specific cysteine proteases (caspases) 3 and 7 through cleavage of the inactive form (procaspase) is involved in the apoptosis process, while the cleavage of poly(ADP) ribose polymerase (PARP) is recognized as the final step of the programmed cell death.

**Figure 6:**
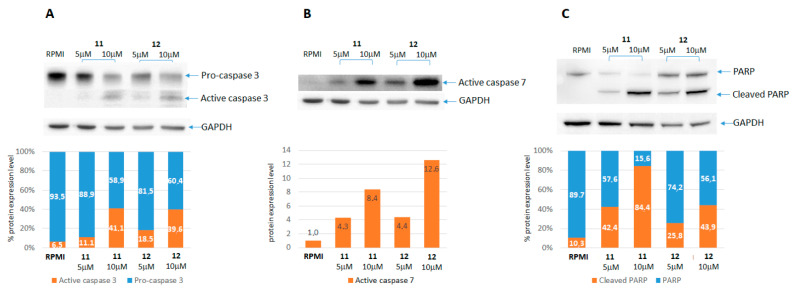
Caspases and PARP cleavage: Western blotting. Cells were treated with 5 and 10 μM of either **11** or **12** for 24 h. Cell lysates were loaded on 4–12% Bis-Tris gel, resolved on SDS-PAGE, and transferred on nitrocellulose filters, as described in the Materials and Methods. Filters were hybridized, respectively, with the following primary antibodies: anti-caspase 3 (panel **A**), anti-caspase 7 (panel **B**), and anti-PARP (panel **C**). The graphs represent the levels of protein expression, quantified by the UVITEC mini-HD6 imaging system, analyzed and normalized against the levels of the GAPDH housekeeping gene expression with UVITEC UVI-D1 software [36].

To better characterize the proapoptotic activity observed by annexin V assays, we performed Western blot assays with the aim to verify if our new compounds could activate apoptosis through the involvement of caspases and PARP. A set of three MM cell lines (CN, SK, and A375) were treated with 5–10 μM of **11** or **12** for 24 h and then tested by Western blotting for caspases 3 and 7 and the PARP enzyme. The exemplificative results obtained from one cell line are reported in Figure 6. These show the ability of the **11** and **12** treatments to dose-dependently activate/cleave both caspases and PARP, confirming the onset of apoptosis, demonstrated during previous experiments. In particular, for caspase 3 (Figure 6A) and the PARP enzyme (Figure 6C), antibodies detected both precursors and active forms, highlighting a gradual decrease of the first (precursor) that corresponds to a progressive increase in the second (active-cleaved) in a dose-dependent manner (Figure 6A,C). For caspase 7, instead, the antibody binds specifically only the active form, confirming a dose-dependent increase in the protein following the **11** and **12** treatments (Figure 6B). All of the three tested cell lines showed highly similar findings, endorsing the evidence of a proapoptotic trend involving caspases and PARP as a consequence of the **11** and **12** actions. Western blot images for all three cell lines are presented in Figure S4 of the Supplementary Materials. The cleavage of caspases 3 and 7 and PARP are events occurring downstream of the phosphatidyl-serine membrane exposure, thus confirming the occurrence of apoptosis and indicating that caspase activation is involved in the process of cell death that was observed.

#### 2.2.3. TUNEL Assays

**Figure 7:**
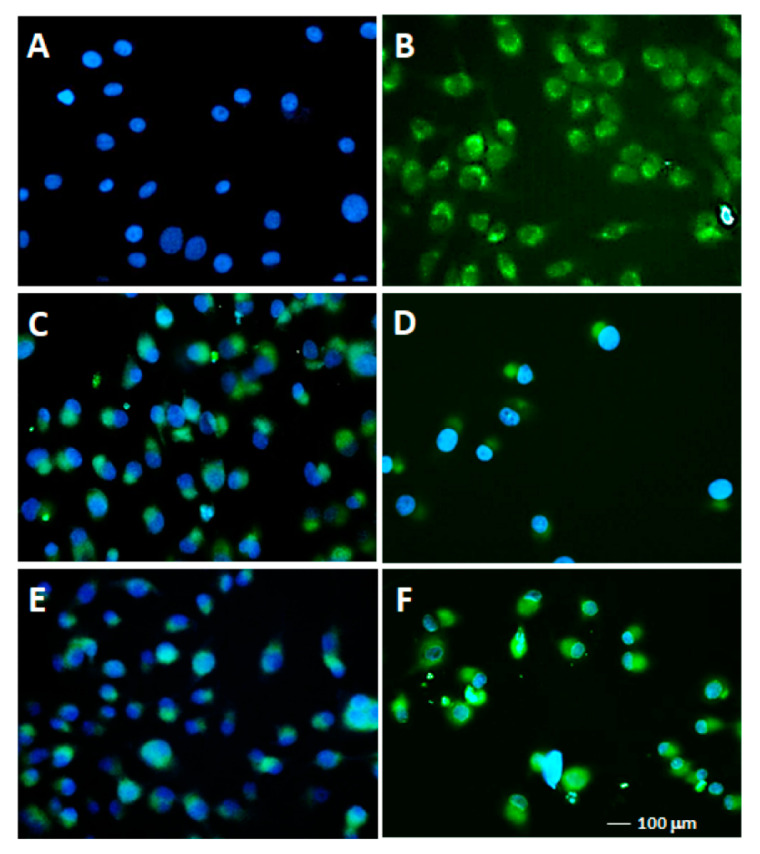
TUNEL assays. To highlight the DNA fragmentation of apoptosis, TUNEL assays were performed on the SK cells, as described in the Materials and Methods. Cells were seeded on chamber slides and then untreated (**A**) or treated with 5–10 μM of either **11** (**C**,**D**) or **12** (**E**,**F**) for 24 h. Samples (**B**) were SK cells treated with TACS nuclease. The slides were observed with the Olympus BX61 fluorescence microscope with a FITC filter, 20× magnification. DAPI (blue fluorescence) was used as the contrast dye to highlight the nuclei.

Finally, we conducted some TUNEL experiments on SK, one of the cell lines that was more sensitive to the proapoptotic activity of **11** and **12**, which also showed a larger morphology. The TUNEL assay highlighted the fragmentation of DNA, resulting in the end of the apoptotic process. The SK cells were treated with 5 and 10 μM of **11** and **12** for 24 h and then processed for the TUNEL assay, as described in the Material and Methods section. As shown in Figure 7, a dose-dependent increase in green fluorescence characterized the cells treated with **11** and **12**. Particularly, the cells treated with 5 μM **11** and **12** showed many fragmented nuclei with the presence of clearly visible green (fluorescein) apoptotic bodies next to the blue (DAPI) nuclei of living cells (Figure 7C,E). The 10-μΜ-treated samples displayed a lower density of the cells, many of which showed green fluorescence due to the undertaken apoptotic process (Figure 7D,F). Such a low cell density was likely due to the advanced process of apoptosis, causing the cells to detach from the slide surface while dying.

### 2.3. Cell Cycle Analysis

**Figure 8:**
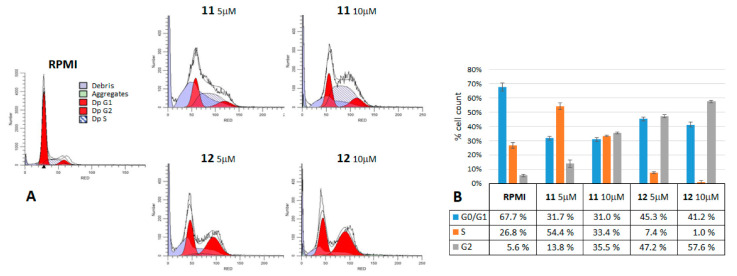
Cell cycle arrest. A375 MM cells were treated with 5 and 10 μM **11** and **12**, for 24 h and then analyzed by Tali^®^ Cell Cycle assays. (**A**) The results were analyzed with ModFit^®^ software [37], and the extrapolated cytograms were reported as exemplificative of a single experiment. (**B**) Histograms represent the percentage of cells in each G0/G1, S, and G2 phase as the mean values of three experiments ± standard deviation.

To determine whether compounds **11** and **12** could interfere with the cell cycle progression, a propidium iodide staining of the treated cells was performed and analyzed using the Tali^®^ cytometer. The MM cells treated for 24 h with 5 and 10 μM of either **11** or **12** were analyzed using the Tali^®^ Cell Cycle kit, as reported in the Materials and Methods. The fluorescence data were elaborated by Modfit LT4.1 software [37], and Figure 8A,B shows the results obtained for the A375 cells as the example better reflecting the behavior of all five MM cell lines. After the **11** and **12** treatments, the peak corresponding to the G1 phase of the cell cycle decreased, compared to that of the control (RPMI), while the S or G2 phase peaks increased, thus suggesting a block in the G2/M transition. Such an increase denoted, indeed, a cell cycle arrest causing an accumulation of cells in the phase of DNA synthesis (S, for **11** treatments) before the completion of the duplication or just after that (G2, for **12** treatments), representing the cells that cannot pass to the mitosis phase (M), because they are blocked at the checkpoint between G2 and M. Our results confirmed that the antiproliferative activity exerted by **11** and **12** contrasting the growth of melanoma cells is related to their ability to interfere with cell cycle progression, causing it to stop in the S/G2 phases and likely taking cells to die by apoptosis.

## 3. Discussion

The therapeutic approach contrasting advanced melanoma, the most aggressive cutaneous tumor, has recently taken an encouraging trend, due to the development of new targeted and immunotherapies [13]. However, it remains the topic of a cure for patients either lacking a *BRAF* mutation or who are refractory to therapies, in addition to those that develop resistance to them [14,16]. The theme of finding new antitumor compounds able to front these issues is not new but still current, and natural compounds and their derivatives have often been investigated in such a direction. Among them, curcumin and its analogs gave promising results, and our group has deeply described the antitumor activity of the curcumin analog D6, also highlighting its possible molecular mechanisms of action [30,31,32]. Our new compounds **11** and **12**, that we have recently described among a group of thirteen hydroxylated biphenyl compounds structurally related to D6 [33] were here investigated with the aim to test the capability of improving the D6 activity against melanoma cells. These compounds combined in a single molecular structure two classes of natural compounds of extreme interest for the design and synthesis of a new pharmacological lead: curcumin and hydroxylated biphenyl. Curcumin, the first one, is a natural, multitarget compound that does not show any toxicity and whose tolerability is almost absolute [22,28]. Compound D6 can be considered a combination of a hydroxylated biphenyl unit and an α,β-unsaturated carbonyl methyl group that forces the curcumin structure to split in two parts and is linked to the aromatic rings (Figure 1). Although, in the literature, the presence of articles and reviews regarding the design, synthesis, and study of curcumin derivatives is extremely nourished [23,24,26,27], our set of compounds represents the only example of curcumin analogs structurally related to the family of hydroxylated biphenyls. The hydroxylated biphenyls are widely diffused in nature, present in the molecules of extreme pharmacological interest (e.g., vancomycin and ellagitannins), and generally show reduced toxicity compared to the corresponding monomers [38]. The biphenyl scaffold could be able to interact with multiple receptors due to its structural characteristics such as controlled flexibility, fully adaptable in virtue of the presence of proper functional groups [39] and adding new clues to the curcumin structure.

According to our previous work, compounds **11** and **12** having a prenylated chain instead of a methyl group, as in D6, showed a higher lipophilicity and stability [33], and these issues could improve their bioavailability, increasing their capability to cross the phospholipid layer of the cell membrane and improving their diffusion [40]. Our dose vs. response assays showed an antiproliferative activity slightly better than that of D6, reaching IC_50_ values around 1.5 μM in the MM cells, which is about seven-fold stronger than that reported for curcumin itself (IC_50_ 10.2 µM ± 1.9; mean value over six MM cell lines, including CN and GR [30]). These are very low IC_50_ values compared with curcumin and other published curcumin analog compounds [23,24,25,27]. Moreover, the wash-out experiments evidenced a lower minimum time of response for **11** and **12** (3 h on CN and A375), with respect to D6 (6 h on CN), and a lower minimum dose of activity in the clonal growth conditions [30]. The antiproliferative activity is the first feature required by a potential anticancer compound, counteracting one of the principal hallmarks of cancer, uncontrolled proliferative signaling [41]. The results obtained from our present work showed that both compounds **11** and **12** accomplish this task. As well, they maintained selectivity against cancer cells, as did D6, normal BJ fibroblast growth not significantly affected by their activity. Uncontrolled cell growth in cancers is largely due to cell cycle dysregulation ending in tumor progression. Our data on the cell cycle analysis by PI staining highlighted the property of **11** and **12** to arrest the uncontrolled advancement of the cell cycle by preventing cells from passing the G2/M checkpoint. A375 cells gave the clearest results in this assay, compared to the other MM cells, probably because of being the most sensitive to the antiproliferative activity of such compounds (Table 1 and Table 2 and Figure 2 and Figure 3). We observed a difference in the behaviors of the two compounds, **11** increasing both the S and G2 phase peaks, while **12** increasing only the G2 phase peak. This evidence could reflect the different timing of the intake and/or reactivity for the two compounds, finally taking both to prevent the cells from entering mitosis. This was another feature of D6 that is maintained by its derivatives **11** and **12**, which effect on cell cycle was mostly in accumulating cells in the G2 phase. Such behavior represents a defense mechanism of the cell, unable to correctly pass the control, which actually causes a block in cell proliferation, and it is highly correlated with the initiation of apoptosis. Indeed, the last, but not least, feature of **11** and **12** is their ability to induce apoptosis on melanoma cells. This is another important property required by a potential anticancer therapeutic agent, to be able to restore defective mechanisms of apoptosis induction [41]. Resistance to apoptosis is typical of melanoma, and its reversal is a common aim of most preclinical combination therapy studies [42]. Here, we obtained evidence that **11** and **12** triggered apoptosis by investigating three sequential hallmarks of this kind of cell death. First, we observed phosphatidylserine exposure on the outreach of the cell membrane by the annexin V assays. Second, we observed the caspase 3 and 7 activations, together with the PARP cleavage. Third, we verified the DNA fragmentation and apoptotic body formation by the TUNEL test. All these methods highlighted an evident apoptosis increase in the MM cells treated with **11** and **12**, while normal fibroblasts were not significantly affected. Apparently, the **11** compound showed a stronger proapoptotic effect than **12** on the MM cell lines. Both the **11** and **12** compounds were shown to induce apoptosis in a stronger and quicker way, compared to D6, reaching even more than 40–60% of apoptotic cells, respectively, after 16 h of 5-μM treatments (see the SK-mel cell line). Instead, D6 was shown to give a similar effect only after 48 h at 10-μM concentration [30].

A hypothesis to explain the faster effects exerted by compounds **11** and **12** with respect to D6, on both apoptosis triggering but, also, on the antiproliferative activity, as discussed above, could be the presence of prenylated chains that might increase their bioavailability and selectivity, possibly inducing quicker molecular interactions inside the cell and a prompt cellular response. Indeed, oxoprenylated phenols have been reported as able to interact with different and selected cell receptors and signal transductors, property that could account for their ability in the regulation of the metabolic processes in both the physiological and pathological conditions [40].

We observed a certain heterogeneity among the different cell lines, with respect to the timing and power of the **11** and **12** effects. Such a variability of response, especially in short-term treatments, was actually expected and could probably be linked to different genetic profiles characterizing the five MM cell lines chosen, representing different molecular types of melanoma [34]. A similar variability was also described for D6 and is a peculiarity of multiple in vitro models. Therefore, we might hypothesize that, initially (T24), the activity exerted by the two compounds **11** and **12** would occur by a variable timing in each cell line (through different targets?), but in the long term (T72), it finally would end in a common mechanism resulting in cell growth inhibition toward all of them. The different molecular signatures could also be an explanation of the diverse behavior with respect to the two principal effects of **11** and **12**: proliferation arrest and apoptosis triggering. As an example, A375 (carrying *CDKN2A*^E61*–E69*^ gene mutations and a *BRAF*^V600E^ gene mutation) were shown to be the most sensitive cell line to the antiproliferative activity of both compounds but, apparently, the least to be stimulated into apoptosis triggering, while SK-mel (carrying a *PTEN*^T167A^ gene mutation) was less affected by the growth inhibition but very much induced to apoptotic death. This variability in response to the intensity for each MM cell line did not affect the significance of our results on the melanoma cells when compared to those observed on normal fibroblasts or those obtained with D6 or curcumin itself. Oppositely, this may highlight the necessity of “targeted treatments” accordingly in the different molecular features among the same tumor (tumor heterogeneity), reflecting the propensity for precision medicine for treating cancer, which is recently gaining ground [43].

Given the structural affinity of **11** and **12** to D6, and their similar effects on melanoma cell viability, growth, and cell cycle progression, we also hypothesized a similar mechanism of action for them. Based on the MM cells expression profile, the D6 effect was indeed speculated to be p53 signaling mediated by the induction of strong cell stress responses contributing to apoptosis stimulation. The down-modulation of several growth signals (c-kit, PI3K/Akt, and NF-kB), as well as the under-expression of cell cycle regulators (cyclin B, cdc25, and CDK4), were also observed, suggesting their involvement in cell growth inhibition [31]. Further investigations are needed to verify whether these new compounds induce similar changes in the molecular profiles of melanoma cells as D6 does to confirm that their mechanism of action involves the modulation of p53 signaling and cell cycle regulators, as suggested by their behavior.

## 4. Materials and Methods

### 4.1. Cell Lines

Five malignant melanoma (MM) cell lines were used as a multiple experimental model for our study. They were chosen among 27 collected in our laboratory as the most suitable to represent the different molecular types of melanoma [34]. They were all obtained from the Istituto Dermopatico dell’Immacolata (IDI, Rome, Italy) and are listed below:

1. CN-mel (CN), derived from melanoma lymph node metastasis, carrying a NRAS^Q61R^ gene mutation;
2. PR-mel (PR), derived from melanoma cutaneous metastasis, carrying a BRAF^V600R^ gene mutation;
3. A375, derived from melanoma cutaneous metastasis, carrying CDKN2A^E61*-E69*^ gene mutations and a BRAF^V600E^ gene mutation;
4. SK-mel (SK), derived from melanoma lymph node metastasis, carrying a PTEN^T167A^ gene mutation;
5. GR-mel (GR), derived from a primary melanoma, carrying a homozygous deletion in the CDKN2B gene.

As a nontumor control, we used a human fibroblast cell line from a healthy donor, the BJ cell line (ATCC^®^, Manassas, VA, USA, #CRL-2522™).

All cell lines were cultured in RPMI medium with stable glutamine, supplemented with 10% fetal bovine serum (FBS) and penicillin/streptomycin (1 U / mL) (all components from Euroclone, Pero, Italy) (complete medium), in a humidified atmosphere with 5% CO_2_ at 37 °C.

### 4.2. Reagents

Biphenyl compounds **11** (3*E*,3′*E*-4,4′-(6,6′-bis(allyloxy)-5,5′-dimethoxy-[1,1′-biphenyl]-3,3′-diyl)bis(but-3-en-2-one)) and **12** (3*E*,3′*E*-4,4′-(5,5′-dimethoxy-6,6′-bis((3-methylbut-2-en-1-yl)oxy)-[1,1′-biphenyl]-3,3′-diyl)bis(but-3-en-2-one)) (Figure 1) were synthesized in our lab, as recently described [33]. Both, prepared as 100-mM stocks in dimethyl sulfoxide (DMSO, Sigma Aldrich, St. Louis, MO, USA), were stored in 50-μL aliquots at −20 °C until use. Compound **11** and **12** working dilutions in complete medium were prepared immediately before use, containing a <0.1% DMSO final concentration.

### 4.3. Cell Proliferation Assays

#### 4.3.1. Dose-Response Assays

Cells were seeded in 96-well plates with RPMI culture medium with stable glutamine, supplemented with 10% fetal bovine serum (FBS) and penicillin/streptomycin (1 U/mL) (complete medium) (all components from Euroclone, Pero, Italy), and grown in a humidified atmosphere with 5% CO_2_ at 37 °C. After 24 h, the medium was replaced by complete medium supplemented or not (control) with increasing concentrations of **11** or **12**. Cells were grown up to 24 or 72 h, and the medium was replaced every 48 h (for 72-h cultures). Cell viability was finally determined by the MTT assay, as described previously [44,45,46]. All experiments were performed in triplicate and repeated at least three times. Relative IC_50_ values were determined by nonlinear regression of the variable slope (four parameters) model by GraphPad Prism software [35]. Statistical significance was determined using the Holm-Sidak method, with alpha = 0.001.

#### 4.3.2. Wash-Out Assays

Wash-out experiments were performed according to the method described by Keshelava [47]. Briefly, cells were seeded on 96-well plates and treated, 24 h later, with 5 μM of either **11** or **12**. After 1, 3, 6, 12, 24, or 72 h, cells were thoroughly washed and then grown in a drug-free, complete medium until a total incubation time of 24 h (T24) or 72 h (T72). Cell viability was determined by the MTT assay, as previously described [46]. The statistical significance of different findings between the experimental groups and controls was determined by the Student’s *t*-test. These findings were considered significant if *p*-values were ≤0.001 or ≤0.05.

#### 4.3.3. Clonogenic Cell Survival Assays

Clonogenic assays were conducted using two experimental conditions: (A) cells growing in liquid medium and treated for 4 h (wash-out) or continuously for 14 days and (B) cells growing in semi-solid media (soft agar) treated continuously for 14 days. MM cell lines were first characterized for their clonal growth capability and optimal seeding density (cells/mL) in both liquid (A) and semi-solid mediums (B). Briefly, sample (A) cells were seeded in a 12-well plate at different concentrations (cells/mL) and grown for 14 days. Then, colonies in each well were stained, counted, and photographed, as described previously [30]; for sample (B), cell suspensions were mixed with 0.3% agarose in RPMI and plated on 2-mm gridded, 6-cm Petri dishes having a bottom layer (0.5% agarose in RPMI medium) freshly solidified. As soon as the top layer became solid, the Petri dishes were incubated at 37 °C in a CO_2_ incubator for 14 days. Colonies inside 10 grids (4 mm^2^ each) were counted through optical inverted microscope observation. The final number, expressing the tumorigenic capacity of each cell line, was elaborated as a percentage of the colonies counted compared to the number of cells initially seeded [48]. Once we established the optimal cell density for each cell line, we proceeded by performing dose/response experiments for both **11** and **12**. Cells grown in liquid medium (A) were treated with 0.5, 1, 2.5, 5, and 10 μM of **11** or **12** for both the wash-out (4 h) and continuous treatment (up to 14 days; treatments every 48 or 72 h), while, for the soft agar experiments (B), the **11** and **12** concentrations used were 1, 2.5, 5, and 10 μM. Colonies were then quantified as described above.

### 4.4. Apoptosis Assays

#### 4.4.1. Annexin V

Annexin V assays were performed using the “Tali ^TM^ apoptosis kit—Annexin V Alexa Fluor^®^ 488 and Propidium Iodide” (Life, ThermoFisher, Waltham, MA, USA) developed as a function of the Tali^®^ image-based cytometer (Life, ThermoFisher), the instrument used to analyze the samples. This assay is based on the detection of the green fluorescent annexin V, labeled with Alexa Fluor 488, bound to phosphatidylserine exposed on the cell membrane of apoptotic cells. Propidium iodide (PI), instead, binds to the exposed DNA of dead cells and is used to discriminate between necrotic (PI-stained, red) and apoptotic cells (green). Apoptosis induction was determined as the percentage of annexin V-positive cells. Cells showing double fluorescence green/red were considered as in the late stage of apoptosis. MM cells and BJ fibroblasts were seeded on 6-well plates and treated with either **11** or **12** (5 and 10 μM) for 16 h. Untreated cells were used as a negative control, while cells treated with 0.25-μM staurosporine as an apoptosis-positive control. A sample analysis was performed following the manufacturer’s instruction and as previously described [46]. The statistical significance of the different findings between the experimental groups and controls was determined by the Student’s *t*-test. These findings were considered significant if *p*-values were ≤0.01.

#### 4.4.2. Terminal Deoxynucleotidyl Transferase-Mediated dUTP Nick End Labeling (TUNEL) Assays

SK-mel cells were plated in 8-well chamber slides (5 × 10^4^/well), cultured for 24 h, and then treated with either **11** or **12** (5 and 10 μM). After further 24 h, the DNA cleavage was assessed by the TACS^®^ 2 TdT-Fluor in situ Apoptosis Detection Kit (Trevigen, Gaithersburg, MD, USA) following the manufacturer’s instructions. The slides were observed using an Olympus BX61 fluorescence microscope (Olympus Corporation, Shinjuku, Tokyo, Japan) equipped with a FITC filter. The positive control (K+) of the kit consisted of cells treated with a TACS nuclease. DAPI (blue fluorescence) was used as the contrast dye to highlight the nuclei.

#### 4.4.3. Western Blot

Cells were plated in T25 tissue culture flasks with complete medium and grown to semi-confluence. Then, the cells were treated for 24 h with a medium containing (or not) either **11** or **12** at 5- or 10-μM concentrations. Cell lysates were prepared and analyzed by Western blotting, as described previously [31], except for the lysis of cells, which was obtained by solving the cell pellets with the RIPA buffer (Life, ThermoFisher, #89900) containing the 1X Protease Inhibitor Cocktail (Sigma, Merck, Darmstadt, Germany, #P8340) and 200-mM NaVO_4_ as an inhibitor of phosphatases by incubation for 30 min on ice. Protein concentration was dosed by the BCA Protein Assay kit (Life, ThermoFisher, #23227). For each sample, 30 μg of total cellular proteins were loaded on NuPAGE 4–12% Bis-Tris gel 1.0 mm × 10 wells (Life Technologies, #NP0321BOX) for SDS-PAGE and then transferred onto a nitrocellulose membrane using the iBlot gel transfer System from Invitrogen (Life ThermoFisher) as previously described [31]. Ten microliters of Novex™Sharp Pre-stained Protein Standard (Life, ThermoFisher, #LC5800) were loaded, usually in lanes 1 and 7 of each gel as molecular weight markers. The membranes were incubated with primary antibodies at 4 °C o/n. Primary antibodies used were anti-PARP (Abcam, Cambridge, UK, #ab32071), anti-caspase 7 (Life, ThermoFisher, #PA1-26433), and anti-caspase 3 (Life, ThermoFisher, #MA1-91636). Anti-GAPDH (Santa Cruz Biotechnology, Dallas, TX, USA, #sc47724) was instead incubated for 1 h at room temperature. Detection was achieved by incubation with either HRP-conjugated anti-mouse (Merck-MilliporeBurlington, MA, USA, #401215) or HRP-conjugated anti-rabbit (Santa Cruz Biotechnology, Dallas, TX, USA, #sc2004) secondary antibodies for 1 h at room temperature. After washing the membrane with 1X TBST (Thermo Scientific #28360), the ECL Advance^TM^ Western Blotting Detection Kit (GE Healthcare, Chicago, IL, USA, #RPN 2135) was used to visualize the immune complexes. Protein levels were quantified by the UVITEC mini-HD6 Chemiluminescence Imaging System (UVITEC, Cambridge, UK) and UVI-D1 Software [36], normalizing (ratio) the GAPDH protein levels used as an internal control.

### 4.5. Cell Cycle Analysis

Cells were seeded on a 6-well plate in complete medium. After 24 h of incubation at 37 °C, cells were treated with 5 or 10 μM of either **11** or **12** for 24 h. Sample analysis was performed using the “Tali^TM^ Cell Cycle Kit” on the Tali^®^ Image-Based Cytometer (Life, ThermoFisher), following the manufacturer’s instruction and as previously reported [46]. Data obtained were processed using the Modfit LT 4.1 Software [37].

### 4.6. Statistical Analysis

Results are expressed as the mean ± standard deviation. Statistical analysis of the proliferation assays (dose response) data was performed with GraphPad Prism version 7.00 for Windows [35]. Relative IC_50_ values were determined by nonlinear regression, inhibitor vs. response, variable slope (four parameters) curve, and significant mean differences between MM cells and BJ fibroblast (not tumor control) by multiple comparisons by the Holm-Sidak method, alpha = 0.001.

For the wash-out experiments and annexin V assays, the statistical significance of different findings between the experimental groups and controls was determined by the Student’s *t*-test. These findings were considered significant if the *p*-values were either ≤0.001 or ≤0.01 or ≤0.05, as reported in the figure/table captions.

## 5. Conclusions

We confirmed that biphenyls **11** and **12** have significant antiproliferative activity [33], comparable but slightly stronger than that of the structurally related D6 compound [30,31,32], on malignant melanoma cell lines. Like D6, compounds **11** and **12** do not significantly influence the growth of normal fibroblasts. Their activity is irreversible and long-lasting, as cell growth was not restored by compound-free medium for up to 14 days. Such properties improved those previously described for compounds D6, likely due to the presence of a prenylated chain instead of a methyl group in the phenolic-OH, as in D6. Taken together, all our findings show hydroxylated-biphenyl–curcumin derivatives as excellent “lead compounds” for the potential development of drugs that may be useful for treating malignant melanoma. In fact, compared to the therapies currently used, these compounds have a great advantage: they exert a broad spectrum of activity, targeting both the mechanisms of tumorigenesis: the stimulation of unlimited cell proliferation and blockage of apoptosis. Compounds **11** and **12** showed themselves capable of inhibiting the first and restoring the second.

## Figures and Tables

**Table 1:** IC_50_ values for **11** and **12**. The concentrations of compounds **11** and **12** that induced a 50% cell growth inhibition (IC_50_) after 24 h (T24) and 72 h (T72) are listed in the table with their respective 95% confidence intervals (CI). Values were estimated by a nonlinear regression of the variable slope (four parameters) model [35].

Cell Line	11				12			
	T24		T72		T24		T72	
	IC_50_	CI 95%	IC_50_	CI 95%	IC_50_	CI 95%	IC_50_	CI 95%
CN-mel	15.5 μM	11.14 to 28.58	1.7 μM	1.63 to 1.75	14.5 μM	6.46 to 10.30	1.5 μM	1.33 to 1.49
PR-mel	7.1 μM	6.55 to 7.79	2.5 μM	2.54 to 2.73	7.7μM	4.93 to 5.60	1.7 μM	1.85 to 2.08
A375	2.9 μM	2.84 to 3.03	1.5 μM	1.27 to 1.36	3.8 μM	2.32 to 3.10	1.4 μM	1.27 to 1.42
SK-mel	7.7 μM	6.91 to 8.84	2.5 μM	2.46 to 2.56	19 μM	7.02 to 18.90	2.2 μM	2.19 to 2.36
GR-mel	6.4 μM	5.86 to 7.06	3.2 μM	2.88 to 3.15	12.3 μM	7.41 to 11.02	3.4 μM	2.75 to 3.28
BJ	>32 μM		6.8 μM	6.50 to 7.26	>32 μM		6.0 μM	5.55 to 6.90

**Table 2:** Clonal growth inhibition. The table lists (**A**) the tumorigenic capacity of each cell line (% clonal growth), representing the percentage of colonies grown in clonal conditions compared to the number of cells initially seeded, and (**B**) the concentrations of **11** and **12** able to totally inhibit the melanoma cells clonal growth in both the semi-solid and/or liquid media culture conditions.

A		B					
MM Cell Line	% Clonal Growth	11			12		
		Liquid Medium		Semi-Solid Medium	Liquid Medium		Semi-Solid Medium
		In Continuum	Wash Out		In Continuum	Wash Out	
A375	4.65	--	--	2.5 μM	--	--	2.5 μM
PR-mel	9.86	--	--	10 μM	--	--	10 μM
CN-mel	9.61	2.5 μM	10 μM	5 μM	1 μM	10 μM	5 μM
SK-mel	7.00	1 μM	5 μM	--	1 μM	5 μM	--
GR-mel	not able to develop clonal growth from single cells, both in the liquid and semi-solid medium						

**Table 3:** Percentage of apoptosis—annexin V. Apoptosis induction was determined as the percentage of annexin V-positive cells. The table reports the percentages of apoptosis induced by 16-h treatments with 5 and 10 μM of either **11** or **12** on malignant melanoma cells (1–5) and normal fibroblasts (6). Values in the table represent the mean percentage of annexin V-positive cells out of three independent experiments ± standard deviation. * *p* ≤ 0.01 (MM (1–5) vs. BJ (6)).

% of Apoptotic Cells						
Cell Line		RPMI	11		12	
			5 μM	10 μM	5 μM	10 μM
1	SK-mel	4.2 ± 0.9	62.6 ± 1.8 *	67.1 ± 1.1 *	42.0 ± 1.2 *	49.3 ± 0.5 *
2	PR-mel	5.5 ± 0.7	17.7 ± 0.9 *	68.6 ± 0.4 *	21.7 ± 0.9 *	33.7 ± 1.7 *
3	CN-mel	2.2 ± 0.4	12.6 ± 0.9 *	31.4 ± 1.1 *	18.2 ± 1.2 *	34.2 ± 0.5 *
4	GR-mel	3.7 ± 1.0	18.1 ± 0.2 *	34.6 ± 1.0 *	6.7 ± 1.1	21.5 ± 2.2 *
5	A375	1.7 ± 0.7	10.3 ± 1.3 *	19.2 ± 1.1	12.3 ± 2.3	18.7 ± 0.7 *
6	BJ	6.1 ± 1.3	5.3 ± 0.8	16.3 ± 3.0	6.9 ± 2.4	4.7 ± 0.6

## Data Availability

The data presented in this study are available within the article and in the Supplementary Figures S1–S4.

## Supplementary Materials

The following are available online at https://www.mdpi.com/article/10.3390/ijms22115636/s1.
