# Supplementary material for: Anticancer Activity of Two Novel Hydroxylated Biphenyl Compounds toward Malignant Melanoma Cells
Source: Int J Mol Sci. 2021 May 26;22(11):5636. doi: 10.3390/ijms22115636 (PMC8198844; doi:10.3390/ijms22115636)
Supplement: Supplementary file 1 [file ijms-22-05636-s001.zip › Captions to Supplementary Figures.pdf]

Captions to Supplementary Figures

Figure S1. Antiproliferative activity of D6, **11** and **12** compared to curcumin. Cells were cultured with increasing concentrations (0,5-10 μM) of curcumin (**A), **D6 **(B), 11, (C**) or **12** (**D**) up to 72 h. Cell proliferation, assessed by MTT assays, was calculated as growth percentages of treated cells compared to the untreated ones (0). Graphs represent the results of three experiments, each done in triplicate, ± standard deviation. ***** $p \leq 0.001$ for data obtained by either D6 or 11 or 12 treatments vs curcumin treatments.

Figure S2. Dose dependent antiproliferative activity of **11** and **12**. Cells were cultured with the indicated concentrations of either **11** (**A, C**) or **12** (**B, D**) up to 24 h (**A, B**) or 72 h (**C, D**). 10 μM cisplatin was used as a positive control for the inhibition of proliferation. Cell proliferation, assessed by MTT assays, was calculated as growth percentages of treated cells compared to the untreated ones (0). Graphs represent the results of three experiments, each done in triplicate, ± standard deviation.

Figure S3. Wash out assays - T24. MM cells and BJ fibroblasts were incubated with 5 μM of either **11** (**A**) or **12** (**B**) for the indicated times, then washed and cultured in drug-free medium up to 24 h (T24). Cell proliferation was assessed by MTT assays as described. Results, derived from three different experiments, each done in triplicate, are expressed as mean percentage of cells growth, ± standard deviation.

° $p \leq 0.05$, MM cell line vs BJ; ***** $p \leq 0.001$, MM cell line vs BJ

Figure S4. Western blotting: Caspases and PARP cleavage on three MM cell lines (CN, SK, A375). Cells were treated with 5 and 10 μM of either **11** or **12** for 24 h. Cell lysates were loaded on 4-12% Bis-Tris gel, resolved on SDS-PAGE and transferred on nitrocellulose filters, as described in Materials and Methods. Filters were sequentially hybridized with the following primary antibodies: anti-caspase 3 (panel **A**); anti-caspase 7 (panel **B**); anti-PARP (panel **C**); anti-GAPDH (panel **D**). The chemiluminescence images were acquired by the UVITEC mini HD6 imaging system. MW: molecular weight markers.
