# Supplementary figures and images for: Anticancer Activity of Two Novel Hydroxylated Biphenyl Compounds toward Malignant Melanoma Cells
Source: Int J Mol Sci. 2021 May 26;22(11):5636. doi: 10.3390/ijms22115636 (PMC8198844; doi:10.3390/ijms22115636)

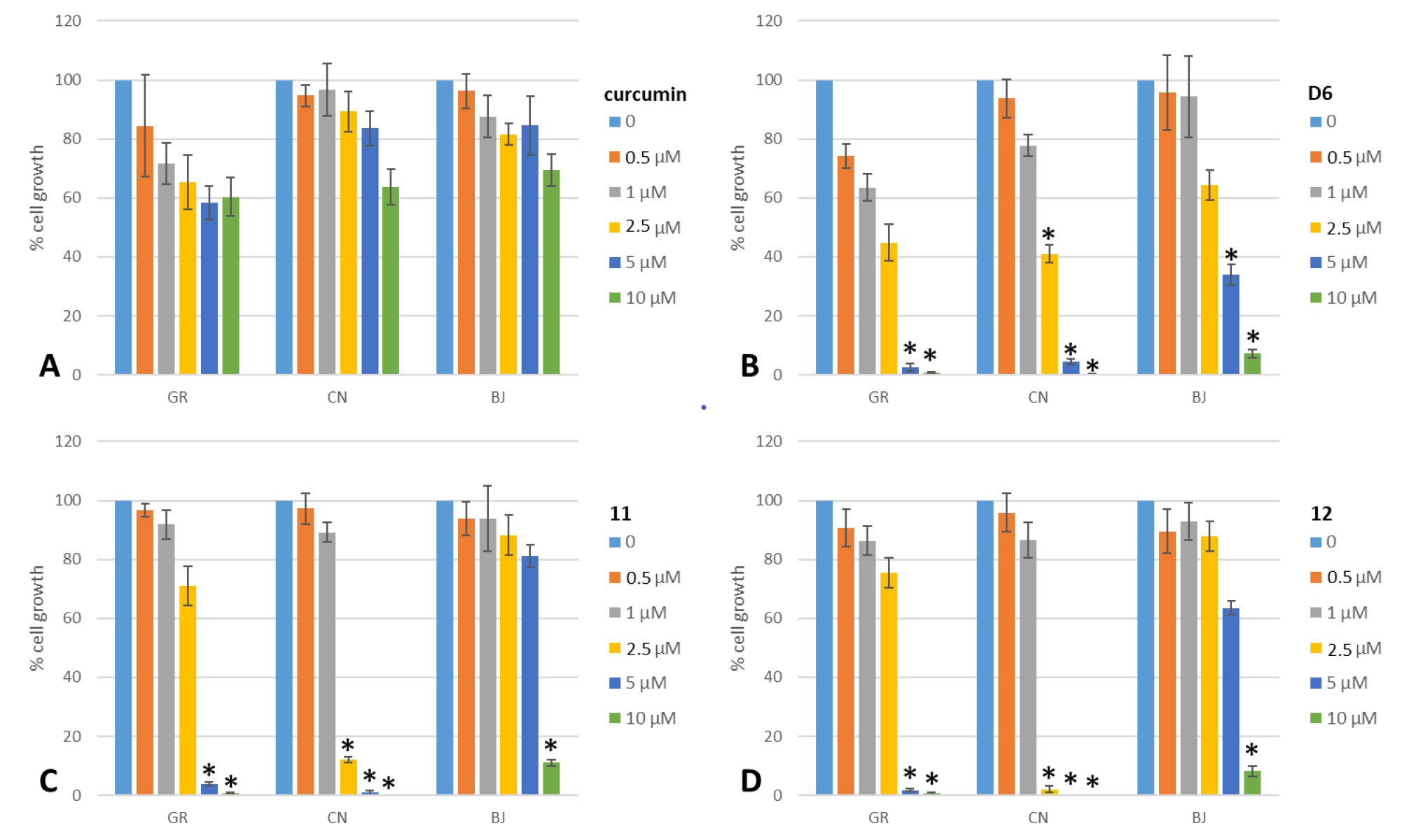

Supplement: Supplementary file 1 [file ijms-22-05636-s001.zip › Figure S1.tiff]

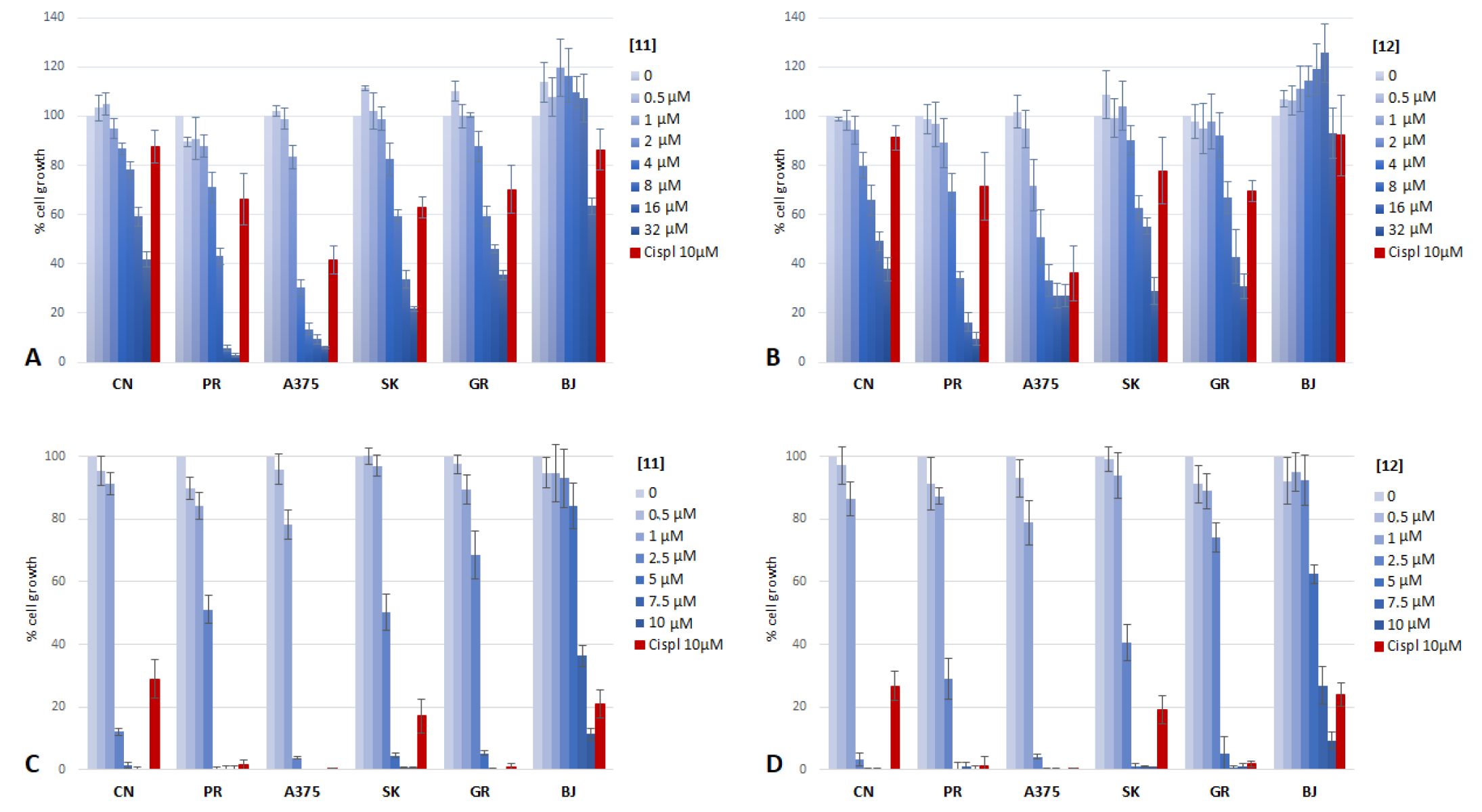

Supplement: Supplementary file 1 [file ijms-22-05636-s001.zip › Figure S2.tiff]

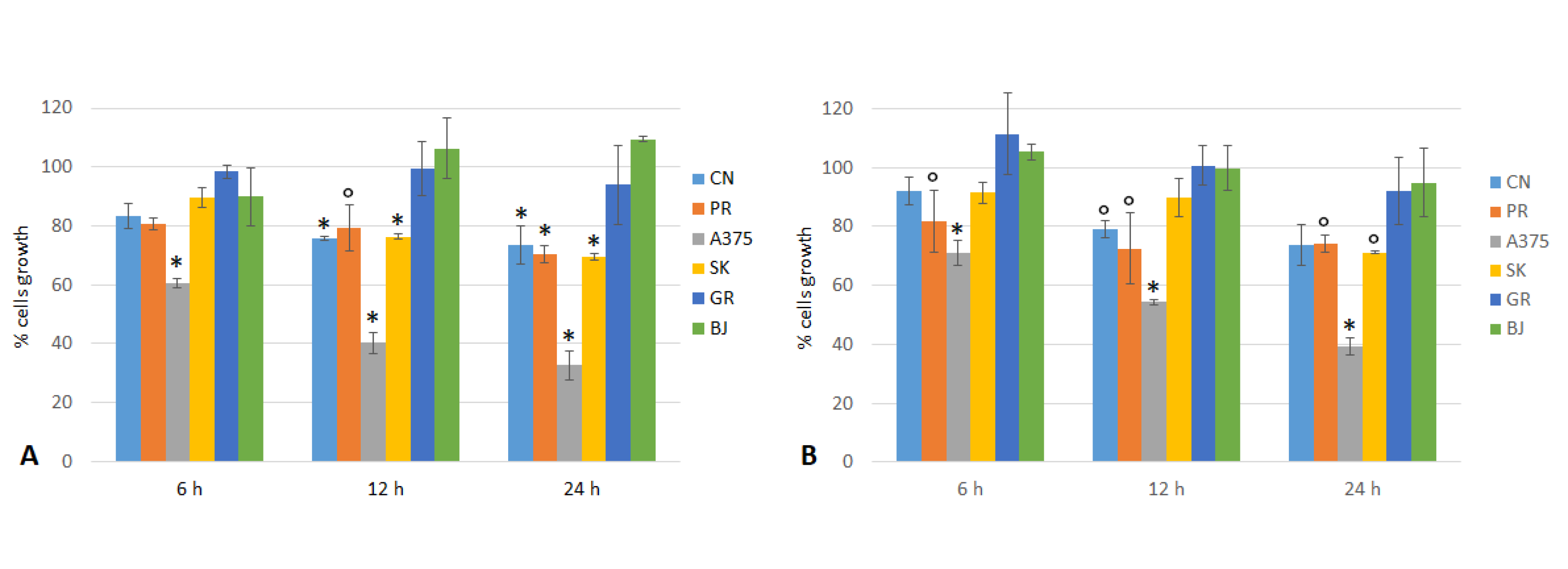

Supplement: Supplementary file 1 [file ijms-22-05636-s001.zip › Figure S3.tiff]

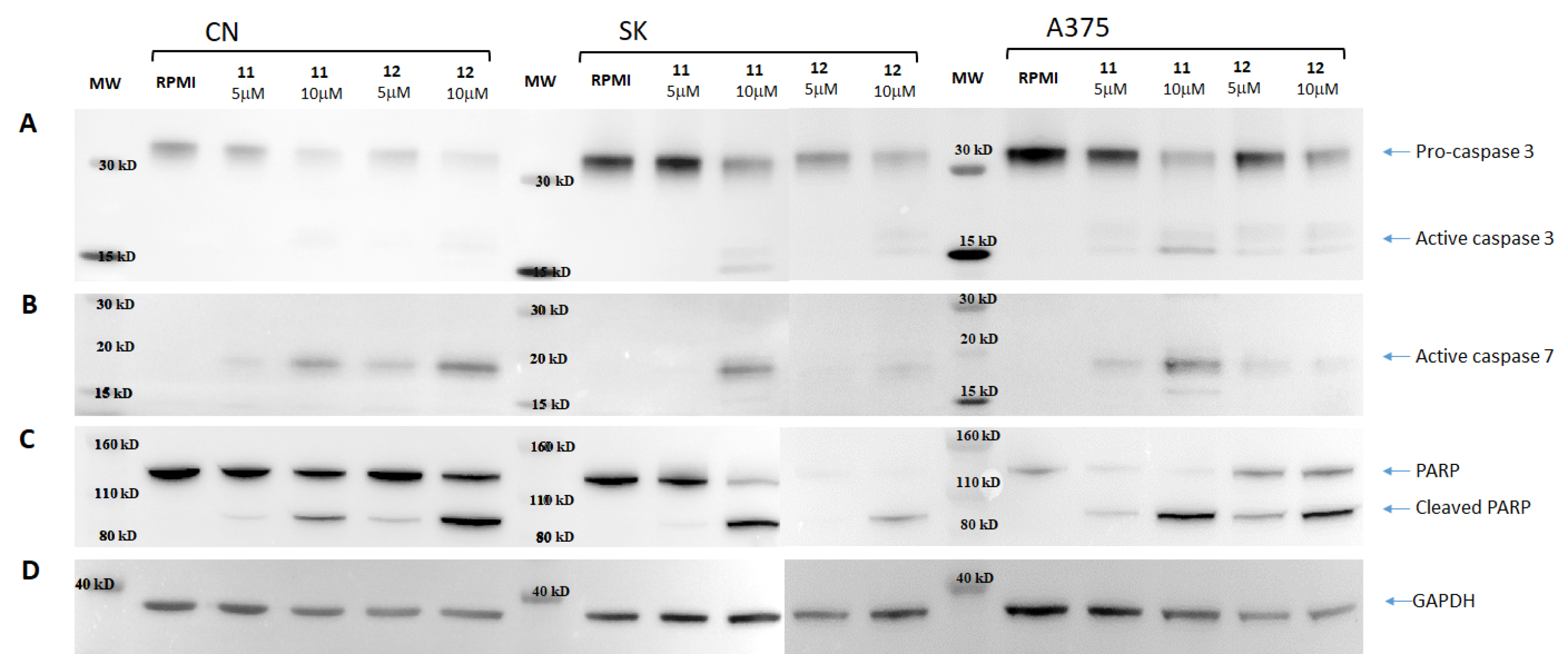

Supplement: Supplementary file 1 [file ijms-22-05636-s001.zip › Figure S4.tiff]
